# Pollinator-mediated selection on flowering phenology and floral display in a distylous herb *Primula alpicola*

**DOI:** 10.1038/s41598-017-13340-0

**Published:** 2017-10-13

**Authors:** Lingling Chen, Bo Zhang, Qingjun Li

**Affiliations:** 1 Key Laboratory of Tropical Forest Ecology, Xishuangbanna Tropical Botanical Garden, Chinese Academy of Science, Mengla, 666303 China; 2 University of Chinese Academy of Sciences, Beijing, 10049 China; 3 Laboratory of Ecology and Evolution Biology, State Key Laboratory in Conservation and Utilization of Bioresources in Yunnan, Yunnan University, Kunming, Yunnan 650091 China; 4 College of Pratacultural Science, Gansu Agricultural University, Lanzhou, 730070 China

**Keywords:** Evolutionary ecology, Experimental evolution

## Abstract

The targets and causes of phenotypic selection are crucial to understanding evolutionary ecology. However, few studies have examined selection quantitatively from multiple sources on the same trait identified the agent of natural selection experimentally. Here we quantified phenotypic selection on traits, including flowering phenology and aspects of floral display via female fitness, in the distylous perennial herb *Primula alpicola*. To determine the role of pollinators in generating selection effects on floral traits, we compared the phenotypic selection gradients in open-pollinated and hand-pollinated plants. Our results show that pollinator-mediated linear selection on flowering start and correlational selection on the number of flowers and scape height explains most of the net phenotypic selection on these traits suggesting pollinators played an important role in shaping floral diversity. We used path analysis and structural equation modeling (SEM) to examine how herbivores affected the relationship between floral traits and female fitness, but no significant selection was caused by seed predators. These results suggest pollinators, not herbivores maybe the significant agent of selection on flora traits.

## Introduction

Explaining the incredible diversity of flowers is a major challenge in evolutionary biology. The major themes in particular, adaptation by natural selection and the benefits of outcrossing^1–3^, have inspired biologists to look for evidence of adaptive evolution in floral function. Identification of the main selective agent is crucial for improving our understanding of floral adaptive evolution. Pollinators and herbivores are traditionally assumed to be the main selective agents^4–7^ and major biological force shaping floral functions such as floral display, morphology and flowering time^8,9^. For example, floral display and morphology not only affect the attractiveness to pollinators and herbivores, but also their behavior^8,10,11^. Flowering time affects the seasonal variation of abundance and behavior of pollinators and herbivores^12–14^. However, most studies quantified the natural selection via single agent^10,12,15,16^, few studies have examined the selection from multiple sources on these traits^11,13,17,18^. We still lack of understanding of mutualists and antagonists corporately in shaping floral evolution.

Applying evolutionary quantitative-genetics theory to the measurement of natural selection^19,20^ and revealing the cause of phenotypic selection on floral traits by manipulating pollination and herbivory environment experimentally have brought great advances in evolutionary biology^11,21^. The relationship between selection patterns and their interactions with pollinators and herbivores has been investigated in many studies^11,15,16^. In many populations of animal-pollinated species, natural selection on floral traits through female function is significantly associated with pollen limitation^22^. Many studies have measured natural selection on floral traits by manipulation of pollen deposition and suggest that pollinators are the agent of natural selection on floral display^16,23^ and flowering phenology^11,21^. However, the agent of selection on flowering phenology and floral display are still rarely identified experimentally^24^. Moreover, selection patterns and agents evidently vary among species, and even among populations and years in the same species^13,15,21,25^. It is not easy to predict the evolutionary effect on floral traits under multiple sources of selection^26^. Therefore, quantified the natural selection by multiple sources and identified the agent of natural selection experimentally could help improve our understanding relative importance of mutualists and antagonists in shaping the plant trait evolution.

*Primula alpicola* is a typical distylous perennial herb and a good material to study floral adaptation and evolution. Distyly is characterized by the reciprocal positioning of stigmas and anthers (long-styled and short-styled morphs), so its reproductive success need to be facilitated by pollinators. From three years continuous field observations at our study site in Lulang (29°33.675′N, 94°44.675′E; 3328 m a.s.l.), Linzhi (Nyingchi), southeastern Tibet, China, we found that pollination of *P*. *alpicola* occurred from May to July and seeds became mature and dispersed from July to August. The major pollinators were bumblebees (*Bombus convexus*), flies, and butterflies. After cultivation of the pre-dispersal seed predators in a cage during fruiting time, we found the larvae of *Amblyptilia punctidactyla* (Pterophoridae, Lepidoptera) fed on the ovules and developing seeds, and often left a hole in ovaries and fruits (L.L Chen, pers. obs.). Different natural selection patterns on floral traits have been documented between tow morphs of *Primula poissonii* ^27^. But the roles of pollinators and predators in natural selection have not been tested in distylous species. In addition, seasonal change of pollinators and predators may affect the natural selection on flowering phenology. In the present study, we therefore attempt to answer the following questions: (1) Whether are the floral traits in *P*. *alpicola* under phenotypic selection in natural population? (2) What are the target traits under selection? (3) Can this selection on floral traits be attributed to the pollinators, the predators, or both?

## Material and Methods

### Study species and site

*P*. *alpicola* is a perennial herb with rosette-forming leaves, an umbellate inflorescence, and fragrant flowers, which are distylous and nearly totally self-incompatible and intra-morph incompatible in both long- and short-styled morphs (L.L Chen, unpubl. data). It is found in wet alpine meadows, especially on stream banks, and open wet areas in forests dominated by trees of Pinaceae, Cupressaceae, Ericaceae, and Fagaceae. It is widely distributed from southeastern Tibet to Bhutan in the Eastern Himalayan region, and especially abundant in valleys, particularly in the Yarlung Zangbo River basin^28^.

Our study population is located in the Sejila (Sygera) Mountains in a typical valley on the northwestern side of the Yarlung Zangbo River (the upper, Tibetan, section of the Brahmaputra). The site has a semi-humid climate, with abundant rainfall brought by the South Asian monsoon, which goes through the Yarlung Zangbo River valley and brings about 71.8% of the annual precipitation from June to September. The mean temperature of the warmest month, July, is 15.8 °C, based on data from the meteorological station in Linzhi (1960–2009)^29^ which is about 30 km from our study site.

### Experimental setup

To quantify natural selection on flowering phenology, we marked plants with one or two opening flowers in a 2 × 2 km study area over a period of 1–2 (−7) days (depending on weather). Each time we marked 60 plants, 40 long-styled and 20 short-styled and then repeated throughout the flowering season from the 155th (June 3) to the 184th day (July 2) after January 1st in 2016. At last 900 plants were marked totally. The marking date was taken as the flowering start time for individuals. Because flower buds and leaves are generated simultaneously when individuals of *P*. *alpicola* enter the reproductive period, this marking procedure could cover the most plant flowering start time in the studying population, thus increased our ability to detect selection on flowering start time.

In every 60 marked individuals, 20 long-styled and 20 short-styled plants were assigned randomly to the open-pollinated treatment (OP), and 20 long-styled plants were assigned to the supplemental hand-pollination treatment (hereafter, hand-pollinated; HP). We did not hand-pollinate short-styled plants because this could not be done without damaging the flower, which may influence seed production. In the hand-pollinated long-styled plants, the stigmas were brushed with mature anthers full of fresh pollens from short-styled flowers. Donor flowers were selected from at least 20 m away from the pollinated plant. Hand pollination was conducted every three or four days, to make sure every flower received supplemental pollen at least once.

Because the *P*. *alpicola* did not distributed uniformly, to evaluate population flowering phenology, we set up three big quadrats (10 m × 10 m) more than 1 km from each other. For easy recording the flowering individuals, within each big quadrat, we chose 16 small quadrats (1 m × 1 m) at least 2 m apart from each other. In these 48 quadrats we recorded flowering plant numbers every 5 days from the 144^th^ day (May 23^th^) to the 199^th^ day (July 17^th^). Individual plants were considered to be flowering from when the first flower opened until the last flower withered.

### Measured traits

Both vegetative and reproductive organs of *P*. *alpicola* keep growing during flowering time. We defined the flowering start for each individual as the day on which the first one or two flowers opened (the marked time). The number of flowers was counted at fruiting time, when both withered, unfertilized flowers and fruits were still on the stalk. The flower size for each individual was determined by calculating the mean corolla diameter of two fresh fully opened flowers with digital calipers. To reduce the bias from the environment covariance between traits and fitness^21,30^, we treated the diameter of the leaf rosette (hereafter, rosette diameter) as a measure of plant condition. Scape height was measured from the ground to the base of the pedicel. To avoid the variation caused by different growing periods, we measured the rosette diameter, the scape height, and the diameter of the corolla 7 days after the flowering start, when most of the flowers of the inflorescence had opened.

We collected fruits of each marked plant two months after the flowering start, when the rind of the capsule turned transparent and the fruits were matured but not dispersed. All the fruits and seeds produced by marked plants were counted. We estimated female fitness as the total number of seeds from each individual, and relative fitness as the individual fitness divided by the mean fitness of the population. Unfertilized flowers and fruits with larvae, frass, or a hole indicating damage by seed predators were recorded. We calculated the proportion of damaged flowers and fruits as the ratio of damaged flowers and fruits to the total flower number per plant. Fruit set was estimated as the number of fruits divided by the number of flowers per plant.

To estimate pollen limitation in *P*. *alpicola*, we paired open-pollinated and hand-pollinated plants with the same number of flowers, rosette diameter (±1 cm), and flowering start (±1 day) (n = 125 replicates). We quantified pollen limitation for the population as the mean of (seeds in plant that received supplemental hand-pollination – seeds in open-pollinated plant)/(seeds in plant that received supplemental hand-pollination)^15^.

### Statistical analyses

To setup the relationships between flowering date and seed herbivory (proportion of damaged flowers and fruits) and fruit set, we chose the best fitted one with ANOVA from linear, quadratic and cubic models fitted to the data separately.

Because of plant losses, we obtained a total sample size of 516 plants (169 short-styled, 182 long-styled in the open-pollinated treatment, and 165 long-styled in the supplemental hand-pollination treatment). The differences between style morphs on plant traits were examined with analysis of variance (ANOVA) or generalized linear models (GLMs). For model selection, we compared the goodness of fit of GLMs based on the nature of the response variable, and that of ANOVA on transformed values. The goodness of fit of generalized linear models (GLMs) was assessed with the Akaike information criterion (AIC). Differences on the flowering start and number of flowers were analyzed with GLMs with a Poisson error distribution, number of seeds per plant was analyzed with GLMs with a negative binomial error distribution, and the proportion of damaged flowers and fruits was analyzed with GLMs with a binomial error distribution. The differences on scape height and flower size, were examined with ANOVA. The differences on rosette diameters could be examined with ANOVA after it was log-transformed for the purpose of normal distribution. There were no significant differences between long-styled and short-styled plants in the open-pollinated treatment tested by ANOVA/GLMs for flowering start (*Z* = −1.043, *P* = 0.297), scape height (*F* = 0.018, *P* = 0.893), rosette diameter (*F* = 0.090, *P* = 0.764), proportion of damaged flowers (*Z* = 1.006, *P* = 0.314), number of flowers (*Z* = 1.183, *P* = 0.237), and number of seeds per plant (*Z* = 0.248, *P* = 0.804), but there was a significant difference for flower size (*F* = 4.989, *P* = 0.026). When we examined differences in flower size between all long-styled (open and hand pollinated) and short-styled plants with ANOVA, and found no significant difference between them (*F* = 1.224, *P* = 0.269). No significant difference on the flowering phenology, floral display and seed production between two morphs, we excluded flower morph from our natural selection analysis on open-pollination and supplemental hand-pollination treatments. To check for possible differences between pollination treatments in flowering start, number of flowers, flower size, scape height, rosette diameter, seeds production and proportion of damaged flowers and fruits, we used similar ANOVA/GLMs as described in the previous part with pollination treatment as an explanatory variable.

We estimated directional (*β*_*i*_), nonlinear (*γ*_*ii*_) and correlational (*γ*_*ij*_) selection gradients using multivariate regression models following Lande and Arnold^19^. Relative fitness and standardized traits (flowering start, number of flowers, flower size, scape height, rosette diameter, proportion of damaged flowers and fruits) values (mean of 0, variance of 1) were calculated separately for plants of each pollination treatment separately. Directional selection gradients (*β*_*i*_) from a linear model included relative fitness as the response variable and the six traits as independent variables. Nonlinear (*γ*_*ii*_) and correlational (*γ*_*ij*_) selection gradients from the quadratic and cross-product terms were estimated from the full multiple regression model. In the multivariate regression models, standardized proportions of damaged flowers and fruits were used as covariates, and their estimated values are not shown in Tables 2–3. In estimating stabilizing or disruptive selection gradients, we doubled the regression of coefficients for the reported *γ*_*ii*_^31^. As multicollinearity is a potential problem in the multiple regression, we computed variance inflation factors (VIFs) for the linear terms. VIFs for the five floral traits selected in the model were <2, indicating multicollinearity is not a serious problem.

We used analysis of covariance (ANCOVA) to test the effect of pollination treatments on linear and full models respectively. The linear model included relative fitness as the response variable and six standardized traits (flowering start, number of flowers, flower size, scape height, rosette diameter, proportion of damaged fruits and flowers), pollination treatment, and trait × pollination treatment as independent variables. The full model included relative fitness as the response variable and the six standardized traits, pollination treatment, trait^2^, pollination treatment^2^, trait × pollination treatment, trait^2^ × pollination treatment^2^, and trait × trait × pollination treatment as independent variables. We quantified pollinator-mediated selection by estimating gradient coefficients of open-pollinated treatment minus supplemental hand-pollination treatment for each trait: *Δβ*_*poll*_ = *β*_*OP*_ − *β*_*HP*_, *Δγ*_*poll*_ = *γ*_*OP*_ − *γ*_*HP*_^15–17^.

To test the relationship between phenotypic traits and damage by pre-dispersal seed predators, we regressed the proportion of damaged flowers and fruits on five traits (flowering start, number of flowers, flower size, scape height, and rosette diameter) using multivariate regression. The results indicated that flowering start and flower size are significantly positively related to the proportion of damaged flowers and fruits (*R* ^2^ = 0.06, *P* < 0.05; Table S1). Because it was difficult to exclude the pre-dispersal seed predators without simultaneously affecting the pollinators, we used path analysis and structural equation modeling (SEM) to test whether the natural selection on the floral traits was mediated by pre-dispersal seed predators or not, based on the plants under the supplemental hand-pollination treatment which excluded the effect from the pollinators. The preliminary multivariate regression analysis in hand-pollination treatment plants with proportion of damaged flowers and fruits as a covariate indicated selection gradients for number of flowers, flower size, and scape height were significant. In addition, flowering start and flower size were significantly related to the proportion of damaged flowers and fruits. We therefore constructed the model including flowering start, number of flowers, flower size, and scape height. In model A, *A*. *punctidactyla* damage did not mediate selection on any trait via relative fitness. In model B, selection on flowering start, number of flowers, flower size, and scape height were mediated by damage. We statistically tested which model provided the better fit to our data by using SEM^32^. A nonsignificant χ^2^ value indicates that a model has no significant difference from the observed correlations in the data and therefore has a good fit. Moreover Akaike’s information criterion (AIC) and loglikelihood value were included to compare models. The model with smaller AIC and loglikelihood absolute value better fits the data.

All analyses, including generalized linear models (GLMs), analysis of variance (ANOVA), multivariate regression models, analysis of covariance (ANCOVA), path analysis and structural equation modeling (SEM) were performed with R 3.2.3 (R Core Team, 2015). Type III sum of squares tests were used for all analyses of linear models (*ANOVA* function of the CAR package^33^). Generalized linear models (GLMs) used the *glm* and *glm*.*nb* function of MASS package, Path analysis and structural equation modeling (SEM) used the *sem* functions of the LAVAAN package^34^.

## Results

### Flowering individuals, fruit set and pre-dispersal seed herbivory along flowering date

**Figure 1:**
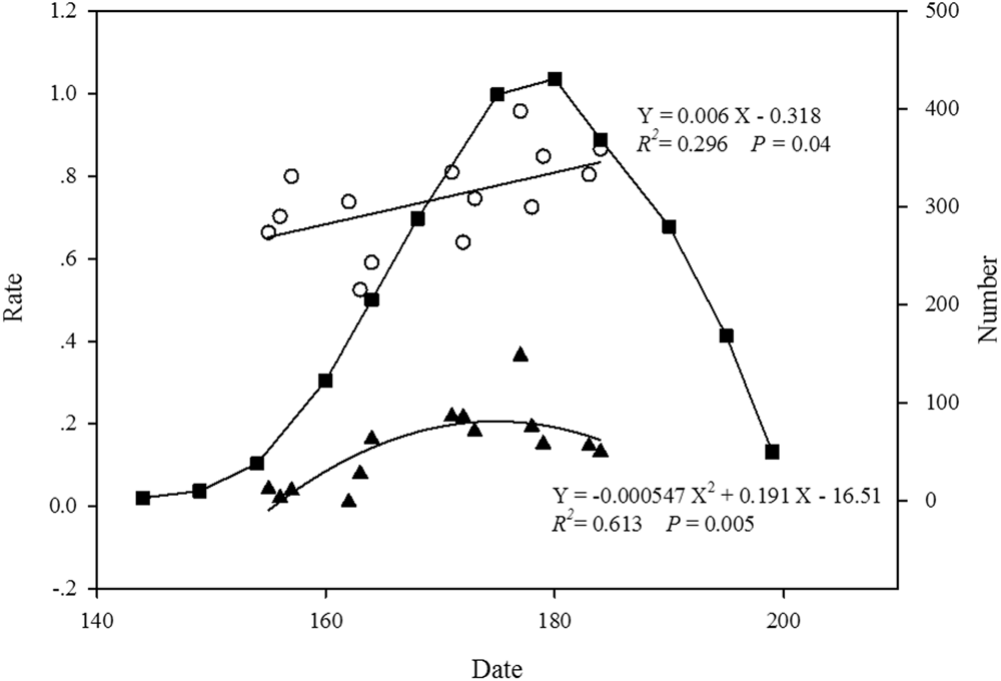
Number of flowering individuals, fruit set (proportion of flowers forming a fruit), and pre-dispersal seed herbivory (proportion of damaged flowers and fruits) with date in one *P*. *alpicola* population at Lulang, China in 2016. -◼- Number of individuals, ▲ Pre-dispersal seed herbivory ○ Fruit set, ---- Fitted curve.

Based on 48 quadrats distributed in 3 plots, the number of plants in flowering were recorded from the 144^th^ day (May 23^th^) and reached the peak of flowering on the 180^th^ day (June 27^th^), and then the number of flowering plants decreased until the 199^th^ day (Fig. 1). Fruit set from the marked plants have significantly increased from the 155^th^ to 184^th^ day following a linear model (*R* ^2^ = 0.296, *P* = 0.04, Fig. 1). Pre-dispersal seed herbivory (The proportion of damaged flowers and fruits) for the marked plants have also significantly increased from the 155^th^ to 184^th^ day following a quadratic model (*R* ^2^ = 0.613, *P* = 0.005, Fig. 1).

### Differences between pollination treatments in floral traits, pre-dispersal seed herbivory, and seed production

There were no significant differences between open- and hand-pollinated treatments in flowering phenology, traits contributing to the floral display, and the proportion of damaged flowers and fruits (Table 1). However, seed production increased significantly after supplemental hand pollination, by 54.7% over open-pollinated plants. The mean pollen limitation was 18.3% (n = 125 replicates) in the population. The mean proportion of damaged flowers and fruits was about 10% in both treatments.

**Table 1:** Flowering phenology, traits contributing to floral display, pre-dispersal seed herbivory (proportion of damaged flowers and fruits), and seed production with means ± SE in both open-pollinated and supplemental hand-pollination treatments in one *P*. *alpicola* population at Lulang, China in 2016.

	Open-pollinated n = 351	Hand-pollinated n = 165	*F* or *Z* value	*P*
Flowering start (day)	168.7 ± 0.538	169.0 ± 0.735	0.272	0.786
No. of flowers	6.8 ± 0.131	6.9 ± 0.194	0.278	0.781
Flower size (mm)	19.6 ± 0.116	19.9 ± 0.181	2.768	0.097
Scape height (cm)	25.1 ± 0.293	25.7 ± 0.465	1.069	0.302
Rosette diameter (cm)	9.7 ± 0.152	9.5 ± 0.201	0.269	0.604
No. of seeds per plant	114.0 ± 5.022	176.2 ± 8.871	**5**.**181**	**<0**.**001**
Prop. of damaged flowers and fruits	0.1 ± 0.012	0.1 ± 0.016	−0.903	0.366

Comparison between pollination treatments was carried out by ANOVA/GLM. Bold *F* or *Z* values and *P*-values indicate significant effects (*P* < 0.05)

### Selection gradients for floral traits

Among open-pollinated plants, Flowering phenology and traits contributing to floral display were subject to significant directional selection (Table 2). There was significant selection for late flowering plants. The production of more flowers was favored in the population. Flower size was subject to positive directional selection. The characters included in the linear model explained approximate half of the variance in fitness among open-pollinated plants (*R* ^2^ = 0.487).

Significant quadratic and correlational selection were detected among open-pollinated plants (Table 2, 3). There was a positive quadratic selection on number of flowers. Late flowering plants with many flowers were favored, as well as plants with combination of late flowering and two other traits (short scape, big rosette diameter). There was negative correlational selection on number of flowers and scape height, and positive correlational selection on flower size and rosette diameter. The characters included in this full model explained more than a half of the variance in fitness among open-pollinated plants (*R* ^2^ = 0.573).

### Evidence for pollinator mediated natural selection

**Figure 2:**
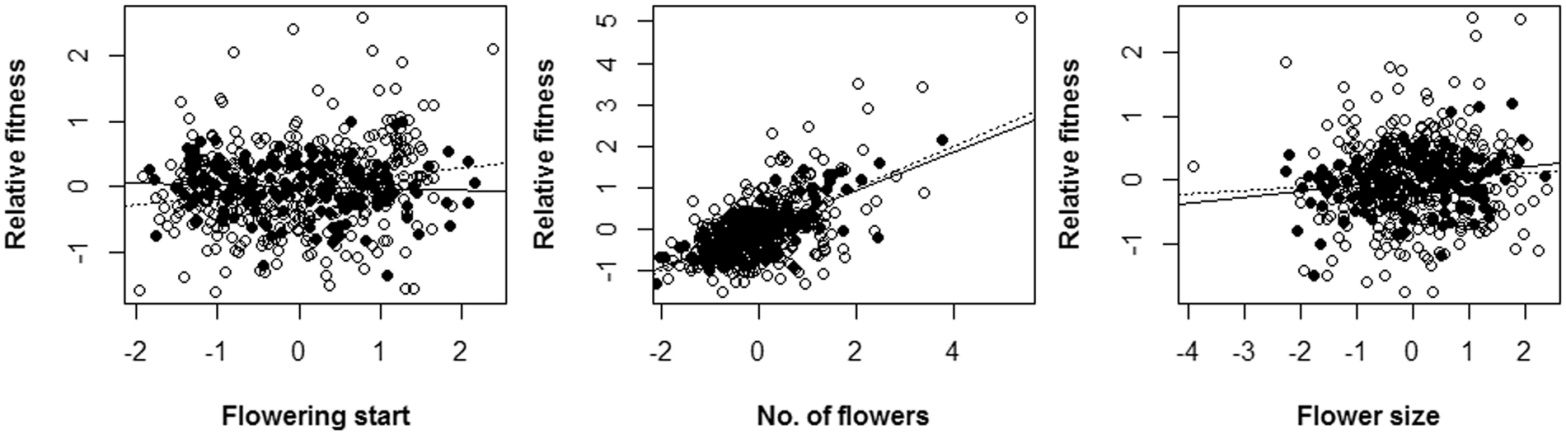
Added-variable plots for the traits flowering start, number of flowers and flower size which were detected significant linear natural selection gradients on among open-pollinated plants in one *P*. *alpicola* population at Lulang, China in 2016. In these added-variable plots, residuals from a linear regression model of relative fitness on all traits except the focal trait are plotted against the residuals from a regression model of the focal trait on the other traits under open-pollinated treatment (open symbols and dashed line) and hand-pollinated treatment (closed symbols and solid line).

Pollinators contributed significantly to directional selection on flowering start and correlational selection on flower number and scape height, and variation in pollinator-mediated selection could explain most of the net phenotypic selection on these trait and trait combination among open-pollinated plants (significant trait × pollination treatment interactions, Table 2; significant trait combination × pollination treatment interactions, Table 3). In open-pollinated plants, there was a significant positive linear selection gradient for flowering start, but in hand-pollinated plants the linear selection gradient was no longer significant. The significant pollinator-mediated selection for late start of flowering (*Δβ*_*poll*_ = 0.206, *P* < 0.05), accounted for all of selection observed among open-pollinated plants (*β*_*i*_ = 0.194) (Table 2; Fig. 2). Correlational selection on number of flowers and scape height differed significantly between open- and hand-pollinated plants. Relative fitness was negatively related to the combination of number of flowers and scape height among open-pollinated plants, but pollen supplementation reversed the direction of correlational selection. Pollinators selected for more flowers and shorter scape plants (*Δβ*_*poll*_ = −0.177, *P* < 0.05), accounting for all of the observed net selection (*β*_*ij*_ = −0.088) (Table 3).

**Table 2:** Linear (*β*_*i*_ ± SE) and quadratic (*γ*_*ii*_ ± SE) selection gradients for open-pollinated (n = 351) and hand-pollinated (n = 165) plants and pollinator-mediated selection gradients with proportion of damaged flowers and fruits as a covariate in one *P*. *alpicola* population at Lulang, China in 2016.

Phenotypic trait	Open-pollinated n = 351		Hand-pollinated n = 165		Pollinator-mediated			
	*β*_*i*_ ± SE (*R* ^2^ = 0.487)	*γ*_*ii*_ ± SE (*R* ^2^ = 0.573)	*β*_*i*_ ± SE (*R* ^2^ = 0.695)	*γ*_*ii*_ ± SE (*R* ^2^ = 0.784)	*Δβ*_*poll*_	*P*	*Δγ*_*poll*_	*P*
Flowering start	**0**.**194** ± **0**.**035**	0.164 ± 0.046	−0.012 ± 0.031	−0.036 ± 0.036	**0**.**206**	**<0**.**001**	0.200	0.360
No. of flowers	**0**.**495** ± **0**.**036**	**0**.**104** ± **0**.**022**	**0**.**445** ± **0**.**032**	0.01 ± 0.023	0.050	0.510	0.094	0.832
Flower size	**0**.**094** **±** **0**.**035**	0.044 ± 0.026	**0**.**111** ± **0**.**030**	−0.014 ± 0.027	−0.017	0.561	0.058	0.152
Scape height	−0.002 ± 0.039	0.054 ± 0.024	**0**.**097** **±** **0**.**033**	**−0**.**17** ± **0**.**031**	−0.099	0.103	0.224	0.557
Rosette diameter	−0.010 ± 0.038	−0.064 ± 0.027	−0.052 ± 0.034	−0.072 ± 0.031	0.042	0.447	0.008	0.935

*Δβpoll* = *β*_*OP*_ − *β*_*HP*_, and *Δγpoll* = *γ*_*OP*_ − *γ*_*HP*_ are pollinator-mediated linear and quadratic selection gradients respectively. *P*-values are associated with differences in selection gradients between pollination treatments (the trait × pollination treatment interaction) in ANCOVA. Significant selection gradient estimates and their *P*-values are indicated in bold (*P* < 0.05).

**Table 3:** Correlational selection gradients (*μ* ± SE) among open-pollinated and hand-pollinated plants and pollinator-mediated selection gradients, with proportion of damaged flowers and fruits as covariate, in one *P*. *alpicola* population at Lulang, China in 2016.

	Open-pollinated *γ*_*ij*_ ± SE	Hand-pollinated *γ*_*jj*_ ± SE	Pollinator-mediated	
	(*R* ^2^ = 0.573)	(*R* ^*2*^ = 0.784)	*Δμ*_*poll*_	*P*
Flowering start × No. of flowers	**0**.**133** **±** **0**.**040**	−0.024 ± 0.038	0.157	0.191
Flowering start × Flower size	−0.003 ± 0.038	0.016 ± 0.029	−0.019	0.453
Flowering start × Scape height	**−0**.**088** **±** **0**.**041**	−0.008 ± 0.038	−0.080	0.190
Flowering start × Rosette diameter	**0**.**073** **±** **0**.**036**	**0**.**097** **±** **0**.**044**	−0.024	0.283
No. of flowers × Flower size	0.052 ± 0.039	0.015 ± 0.033	0.037	0.547
No. of flowers × Scape height	**−0**.**088** **±** **0**.**040**	**0**.**089** **±** **0**.**035**	**−0**.**177**	**0**.**005**
No. of flowers × Rosette diameter	0.041 ± 0.045	−0.042 ± 0.043	0.083	0.392
flower size × Scape height	0.003 ± 0.045	**−0**.**073** **±** **0**.**035**	0.076	0.351
flower size × Rosette diameter	**0**.**101** **±** **0**.**043**	**0**.**114** **±** **0**.**039**	−0.013	0.910
Scape height × Rosette diameter	−0.051 ± 0.039	0.047 ± 0.032	−0.098	0.104

Pollinator-mediated selection *Δμ*_*poll*_ = *γ*_*OP*_ − *γ*_*HP*_ and *P*-values associated with differences in selection gradients between pollination treatments (the trait × pollination treatment interaction) in ANCOVA. Significant selection gradient estimates and their P-values are indicated in bold (*P* < 0.05).

### Natural selection by pre-dispersal seed predators

**Figure 3:**
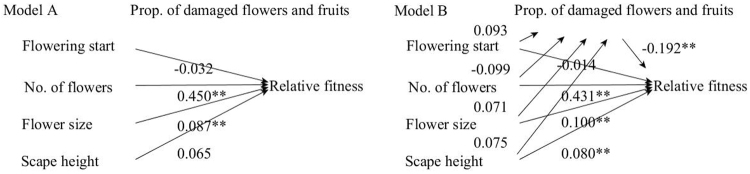
Path analysis of the effect of standardized flowering start, and floral display traits (No. of flowers, flower size, scape height) and proportion of damaged flowers and fruits on relative fitness of *P*. *alpicola* plants under supplemental hand pollination treatment (n = 165) at Lulang, China in 2016. Model A: Four floral traits play direct effects on relative fitness (χ^2^ = 153.3, df = 4, *P* < 0.01, AIC = 2007.7, loglikelihood = −998.8); Model B: Four floral traits play indirect effects on relative fitness through the proportion of damaged flowers and fruits (χ^2^ = 198.5, df = 9, P < 0.01, AIC = 2441.7 loglikelihood = −1209.8). Significant path coefficients are signed with asterisks (*P < 0.05, **P < 0.01).

There was no significant change in selection target between the multivariate regression analyses with and without proportion of damaged flowers and fruits as a covariate. Moreover, we got two alternative, nested models based on the path analysis and structural equation modeling. In Model A, only number of flowers and flower size played significant direct effects on relative fitness (χ^2^ = 153.3, df = 4, *P* < 0.01, AIC = 2007.7, loglikelihood = −998.8). In Model B, no significant indirect selection mediated by pre-dispersal seed predators was detected on flowering start, number of flowers, flower size, and scape height (χ^2^ = 198.5, df = 9, *P* < 0.01, AIC = 2441.7, loglikelihood = −1209.8) (Fig. 3). Model A with a smaller AIC and loglikelihood absolute value better fits the data than model B. These results suggest seed predators did not exert selection on floral traits of *P*. *alpicola*.

## Discussion

This study demonstrates that the pollinators contribute to selection via female fitness on flowering phenology and floral display in the inter-morph pollination distylous perennial herb *P*. *alpicola*. Pollinators mediated directional selection favors late flowering, and correlational selection favors many flowers combined with short scape, that maybe attribute to stronger pollen limitation in the early season and resource tradeoff between pollinators’ attraction and flower production.

Evolutionary ecologists have hypothesized that pollinator-mediated selection should often favor peak or earlier flowering plants^4^, but we found that late flowering plants were favored in *P*. *alpicola*. Several studies have documented early flowering was predominantly favored^35,36^, and people have explained many possible reasons for this phenomenon, from plant characters and environmental factors, such as size, flowering duration, growing season length^37^. But no significant pollinator-mediated selection on phenology was detected in most experimental quantified studies^15,21,38^. The present study demonstrate that natural selection for late flowering plants maybe the adaption to pollinators by seasonal change. Pollinator availability and pollination efficiency can vary during the growing season^39^. Late flowering plants received high pollinator service and high fruit set^40,41^. For *P*. *alpicola*, because of the characters of self-incompatibility and inter-morph pollination, higher pollinator service in late season result in higher female fitness, suggesting that the abundance of the pollinators is a limitation for the relative fitness in the early season in *P*. *alpicola*. Pollinator-mediated selection for late flowering plants may not be uncommon. Natural selection favors late flowering plants attributing to pollinators that also has been reported in *Gymnadenia conopsea* ^11^. Our study may indicate the seasonal pollinator dynamics plays an important role in shaping flowering phenology. This study adds the evidence of biotic interactions in shaping flowering phenology.

Positive correlational selection on number of flowers and plant height has been documented because of synergistical effect of these two traits in pollinator attraction^15^, but to the best of our knowledge this is the first study to demonstrate negative correlational selection on this pair of trait attributing to pollinators. Female fitness is predicted to be simultaneously limited by pollen and reproductive effort (the proportion of a plant’s total resources allocated to reproduction) over a plant’s lifetime^22,42,43^. Reproduction investment involves not only the production of flowering and fruiting structures but also the production of stem material^44^. In present study, pollinators mediated correlational selection favors individuals with more flowers and shorter scapes, suggesting a possible trade-off for resource distribution between pollinator attraction (scape height) and flower production (flower numbers). This is also consistent with the observation that the short-scaped morph had more flowers in *Primula farinose* ^45^. Moreover, in the field, we did not observe any scape height bias for pollinators (L.L Chen, pers. obs.).

Seed predators (*A*. *punctidactyla*) are not likely to have affected selection on flowering phenology and floral display in this study population, although fruit and flower damage reduced relative fitness. The proportion of damaged flowers and fruits was about 10% in *P*. *alpicola*, whereas more damaged fruits (about 21%) was recorded in *Lobelia siphilitica* which had a detectable selection on floral traits via seed herbivores^13^. Many species have documented maybe it was difficult to detect herbivores mediated selection in a low damage level^11,21^. In addition, Seed predators mediated natural selection have been detected on flowering phenology, inflorescence height and flower number, but varied among populations and years^11,46^. These studies suggest fluctuation natural selection may relate to the dynamic intensity of seed predators.

In this study, we experimentally assessed phenotypic selection on floral traits mediated by pollinators in distylous *P*. *alpicola* in the Eastern Himalayan region. We found that pollinators rather than pre-dispersal seed predators mediated directional selection via female fitness on flowering phenology and correlational selection on pairs of traits involved in flowering display. These results add to the growing body of evidence that pollinators are the important force in shaping flowering traits, and improves our understanding of floral diversity in the Eastern Himalayan region. Pollinator-mediated linear selection explained most of the net phenotypic selection on flowering start time, suggesting that pollen limitation was correlated with flowering phenology. Our inability to manipulate the seed predator *A*. *punctidactyla* could have influenced our result, since we were unable to test whether selection by pollinators would have differed if seed predators had been excluded. In ongoing work, we will examine the selection mediated by pre-dispersal seed predators experimentally and pay more attention to the relationships among the population dynamics of flowering plants, pollinators, and pre-dispersal seed predators because of temporal and spatial variation.

## Electronic supplementary material

Supplementary information
