# Author Correction: Pollinator-mediated selection on flowering phenology and floral display in a distylous herb *Primula alpicola*

**DOI:** 10.1038/s41598-019-43381-6

**Published:** 2019-05-02

**Authors:** Lingling Chen, Bo Zhang, Qingjun Li

**Affiliations:** 1 Key Laboratory of Tropical Forest Ecology, Xishuangbanna Tropical Botanical Garden, Chinese Academy of Science, Mengla, 666303 China; 2 University of Chinese Academy of Sciences, Beijing, 10049 China; 3 Laboratory of Ecology and Evolution Biology, State Key Laboratory in Conservation and Utilization of Bioresources in Yunnan, Yunnan University, Kunming, Yunnan 650091 China; 4 College of Pratacultural Science, Gansu Agricultural University, Lanzhou, 730070 China

Correction to: *Scientific Reports* 10.1038/s41598-017-13340-0, published online 13 October 2017

This Article contains errors.

In Figure 1 the two fitted curves should be shown as dashed lines. The correct Figure 1 appears below.

**Figure 1:**
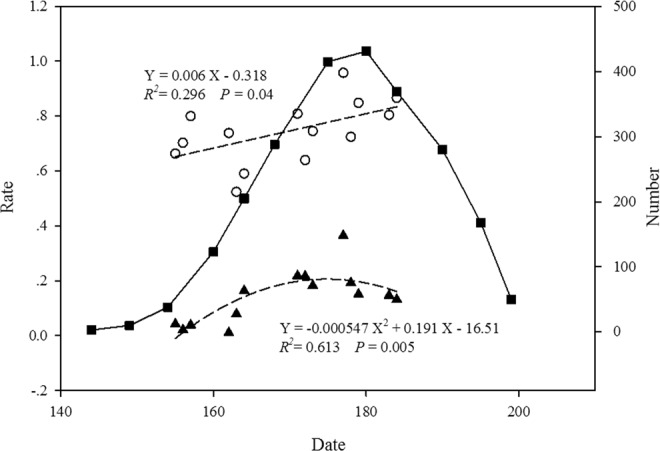
Number of flowering individuals, fruit set (proportion of flowers forming a fruit), and pre-dispersal seed herbivory (proportion of damaged flowers and fruits) with date in one *P. alpicola* population at Lulang, China in 2016. -■- Number of individuals, ▲ Pre-dispersal seed herbivory ○ Fruit set, ---- Fitted curve.

In addition, in the Results section, under the subheading ‘Evidence for pollinator mediated natural selection’,

“Pollinators selected for more flowers and shorter scape plants (*Δβ*_*poll*_  = −0.177, *P* < 0.05), accounting for all of the observed net selection (*β*_*ij*_  = −0.088) (Table 3).”

should read:

“Pollinators selected for more flowers and shorter scape plants (*Δγ*_*poll*_ = −0.177, *P* < 0.05), accounting for all of the observed net selection (*γ*_*ij*_ = −0.088) (Table 3).”

Furthermore, the title of Table 3,

“Correlational selection gradients (*μ*  ± SE) among open-pollinated and hand-pollinated plants and pollinator-mediated selection gradients, with proportion of damaged flowers and fruits as covariate, in one *P*. *alpicola* population at Lulang, China in 2016.”

should read:

“Correlational selection gradients (*γ*_*ij*_ ± SE) among open-pollinated and hand-pollinated plants and pollinator-mediated selection gradients, with proportion of damaged flowers and fruits as covariate, in one *P*. *alpicola* population at Lulang, China in 2016.”

The legend of Table 3,

“Pollinator-mediated selection *Δμ*_*poll*_  = *γ*_*OP*_ − *γ*_*HP*_ and *P*-values associated with differences in selection gradients between pollination treatments (the trait × pollination treatment interaction) in ANCOVA. Significant selection gradient estimates and their P-values are indicated in bold (*P* < 0.05).”

should read:

“Pollinator-mediated selection *Δγ*_*poll*_  = *γ*_*OP*_ − *γ*_*HP*_ and *P*-values associated with differences in selection gradients between pollination treatments (the trait × pollination treatment interaction) in ANCOVA. Significant selection gradient estimates and their *P*-values are indicated in bold (*P* < 0.05).”

Finally, in Table 3, in the ‘Pollinator-mediated’ column,

“*Δμ*_*poll*_”

should read:

“*Δγ*_*poll*_”

