# Supplementary material for: Pollinator-mediated selection on flowering phenology and floral display in a distylous herb Primula alpicola
Source: Sci Rep. 2017 Oct 13;7:13157. doi: 10.1038/s41598-017-13340-0 (PMC5640686; doi:10.1038/s41598-017-13340-0)
Supplement: Supplementary file 1 — Supplementary information [file 41598_2017_13340_MOESM1_ESM.pdf]

Pollinator-mediated selection on flowering phenology and floral display in a distylous herb *Primula alpicola*

Lingling Chen[1,2], Bo Zhang[4], Qingjun Li[3]*

Table S1. The relationship between phenotypic traits and damage by pre-dispersal seed predators by using multivariate regression. Bold *P*-values indicate significant effects (*P*<0.05)

	Flowering start	No. of flowers	Flower size	Scape height	Rosette diameter
Estimate	0.193	-0.011	0.153	0.091	-0.058
SE	0.056	0.058	0.057	0.063	0.062
P	**<0.001**	0.846	**0.007**	0.151	0.349
